# Defusing COVID-19: Lessons Learned from a Century of Pandemics

**DOI:** 10.3390/tropicalmed5040182

**Published:** 2020-11-30

**Authors:** Graciela Mujica, Zane Sternberg, Jamie Solis, Taylor Wand, Peter Carrasco, Andrés F. Henao-Martínez, Carlos Franco-Paredes

**Affiliations:** 1 School of Medicine, University of Colorado, 13001 E 17th Pl, Aurora, CO 80045, USA; graciela.mujica@cuanschutz.edu (G.M.); zane.sternberg@cuanschutz.edu (Z.S.); Jamie.solis@cuanschutz.edu (J.S.); taylor.wand@cuanschutz.edu (T.W.); 2 International Association for Immunization Managers, Washington, DC 20037, USA; rcphs@aol.com; 3 Department of Immunization and Vaccines, World Health Organization, 1202 Geneva, Switzerland; 4 Department of Medicine, Division of Infectious Diseases, Anschutz Medical Center, University of Colorado, Aurora, CO 80045, USA; andres.henaomartinez@cuanschutz.edu; 5 Hospital Infantil de México, Federico Gomez, México City 06720, Mexico

**Keywords:** pandemic, COVID-19, coronavirus, SARS-CoV-1, SARS-CoV-2, MERS, SARS, influenza

## Abstract

Amidst the COVID-19 global pandemic of 2020, identifying and applying lessons learned from previous influenza and coronavirus pandemics may offer important insight into its interruption. Herein, we conducted a review of the literature of the influenza pandemics of the 20th century; and of the coronavirus and influenza pandemics of the 21st century. Influenza and coronavirus pandemics are zoonoses that spread rapidly in consistent seasonal patterns during an initial wave of infection and subsequent waves of spread. For all of their differences in the state of available medical technologies, global population changes, and social and geopolitical factors surrounding each pandemic, there are remarkable similarities among them. While vaccination of high-risk groups is advocated as an instrumental mode of interrupting pandemics, non-pharmacological interventions including avoidance of mass gatherings, school closings, case isolation, contact tracing, and the implementation of infection prevention strategies in healthcare settings represent the cornerstone to halting transmission. In conjunction with lessons learned from previous pandemics, the public health response to the COVID-19 pandemic constitutes the basis for delineating best practices to confront future pandemics.

## 1. Introduction

Throughout the history of mankind, pandemics have consistently produced large-scale demographic, economic, and political disruptions [1,2]. Due to their unpredictable course, fear and anxiety amplify the overall impact of pandemics. Globally, we have experienced multiple waves of the spread of the coronavirus associated disease 2019 pandemic (COVID-19), which is caused by the novel coronavirus SARS-CoV-2 [3]. By November 2020, this pandemic has reached every corner of the planet, and similar to previous pandemics, it has caused substantial medical, economic, and social disruption leading to almost 58 million cases and more than one million deaths [4]. Modern-day experience with pandemics has combined a myriad of public health responses in an attempt to suppress these rapidly evolving pathogens during an era of increased globalization and international travel. While there is a large amount of uncertainty surrounding COVID-19, we have blueprints of similar widespread events that have occurred in the past, particularly the Black Death in the Middle Ages and many influenza pandemics [2]. Unfortunately, pandemics are becoming much more common compared to previous centuries, as shown by the proximity of the 2003 SARS pandemic, the influenza H1N1pdm2009, Chikungunya in 2014, Ebola from 2014–2015, and Zika in 2015. In this narrative review, we compare salient epidemiological aspects of the major influenza and coronavirus pandemics of the 20th and 21st century to identify potential patterns and lessons learned that may assist us in mitigating the impact of the current COVID-19 pandemic.

## 2. Emergence of the Third Coronavirus in the 21st Century

In November 2002, the severe acute respiratory syndrome (SARS) emerged in Southern China. This event marked the documented coronavirus emergence that spread through 20 countries, causing approximately 8000 cases and 800 deaths by producing viral pneumonia and respiratory failure (fatality rate of 9.5%). Traditional public health actions used to control the SARS pandemic included active case detection, isolation of cases, contact tracing with the isolation of cases, social distancing, and community quarantine. Since most of the transmission of SARS-CoV-1 occurred in healthcare settings, the implementation of effective infection prevention interventions resulted in the interruption of clusters of transmission by July 2003. Similarly, the human case of the Middle-East respiratory syndrome (MERS) appeared in Jordan in April 2012 and Saudi Arabia in September 2012, associated with important transmission in healthcare settings and a case-fatality rate of 34%. The COVID-19 pandemic has caused unprecedented medical, social, and economic turmoil that is only comparable to the 1918–1919 influenza pandemic and serves as a stark reminder of how pandemics have molded and marred the evolution and history of humanity [2]. The SARS-CoV-2 coronavirus has been circulating for more than 11 months since it was initially identified in China’s Hubei province in December 2019 [3]. Some of the countries that were most heavily affected by the initial spread of COVID-19 are now seeing new waves of transmission with potentially catastrophic consequences in regions of the Northern Hemisphere entering the winter season [4].

The emergence of zoonotic infections capable of infecting humans has been a relatively common occurrence throughout history. The rate at which these infections cross the animal-human barrier has risen in the 21st century due to increased human-animal contact, habitat destruction, the industrial model of agriculture, and population growth [5]. The transmission has been facilitated by factors such as globalization and ease of travel. This context is important when examining the potential for SARS-CoV-2 to spread across multiple species and generate similar reservoirs. While SARS-CoV-1 and SARS-CoV-2 have proven animal origins, the prevailing theory is that both strains emerged in settings that allowed for a variety of animals to come into close contact with each other [6]. Evidence points to the fact that both coronaviruses were originally harbored in horseshoe bats; however, the identification of an intermediate host remains elusive [7,8]. There is evidence that SARS-CoV-1 is able to be spread to domestic cats, ferrets, and even macaque monkeys. With this knowledge and the growing evidence that SARS-CoV-2 could be highly transmissible to both cats and ferrets, the possible reservoirs for future spread are exponentially larger than was initially considered and across many continents [6,9,10].

The antigenic novelty of a pandemic virus or antigenic shift from a previous pandemic virus can profoundly influence pandemic severity across different regions, populations, and points in time. Indeed, an influenza pandemic arose when novel influenza A viruses containing a new hemagglutinin surface protein subtype present in viral strains derived from waterfowl or swine spread among immunologically naïve human populations. Furthermore, the underlying degree of immunological exposure can play an important role in the impact of a pandemic. This was exemplified during the H2N2 influenza pandemic of 1957, where genetic reassortment into the H3N2 strain was then responsible for the subsequent 1968 pandemic. The retained neuraminidase (N2) surface antigen in this recombination event conferred partial immunity to the pandemic in 1968–1969, particularly in the European and Asian countries that were heavily impacted by the pandemic in 1957 [11]. The partial immunity these areas gained in 1957 is thought to have contributed to the relatively mild first wave they experienced 10 years later. However, many of these areas were also impacted by an H3 antigenic shift that occurred mid-pandemic. This shift is thought to be a driver for the high number of deaths experienced during the second wave in Europe and Asia (70% of all deaths). This pattern was very different in North America, where 70% (USA) and 54% (Canada) of deaths occurred during the first, larger wave of the early fall, theorized to be partially due to the lack of partial immunity conferred from the 1957–1958 H2N2 pandemic [12]. In the case of SARS-CoV-2 infection, there is evidence that some cross-reactive immunity may be conferred by the long-term circulating coronaviruses associated with the common cold (CoV HKU1, CoV NL63, CoV OC43, and CoV 229E). While the degree of immunity conferred by coronaviruses is thought to be short-lived, it may affect the severity, age distribution, and geographic transmissibility of COVID-19 [13].

Mutations of the novel strain within the pandemic period can also influence which waves have the highest case fatality and affect differences in wave transmission patterns by country. An example of mutations occurring throughout multiple seasons of a pandemic took place during the 1918 H1N1 pandemic, where the first wave occurred quite early in June and July in Europe and North America. While there is fragmentary data on the sequence of the virus present in the first wave, we do know from samples that there were strains of the H1N1 virus present during the second wave. However, with the case of 1918–1919, there was conflicting evidence on the influence these H1N1 mutations had on mortality and transmissibility [14]. These mutations go both ways, as seen in the 2004 resurgence of SARS-CoV-1 in South Korea, where a mutation had occurred after the first wave/emergence of SARS. The mutation decreased the S2 protein to the ACE-2 receptor, resulting in decreased transmissibility, disease severity, and mortality compared to the original SARS-CoV-1 sequence [15].

Another inter-pandemic variable is cross-protection, or the number of people exposed who will have immunity in subsequent waves. The basic concept being the relative steps to herd immunity in each wave. In the 1918 influenza pandemic, both the US and British military bases exposed to the first wave provided a 35–94% protection against severe symptoms and 56–89% protection against death during the second wave [16,17,18]. This strategy was recently employed in Sweden, where lockdowns were minimized compared to most countries facing COVID-19 with the expectation that herd immunity would be reached faster to minimize further waves of transmission. It should be stated that this is not a realistic approach for many countries as it works best when a country has a certain age demographic (younger and healthier populations) and a healthcare system capable of handling a large number of cases.

During the COVID-19 pandemic, case isolation and tracing of contacts have given way to mitigation interventions attempting to reduce the number of hospitalization and fatalities. Indeed, the SARS-CoV-2 coronavirus has shown to be efficiently transmitted within households and in the community and with a significant amount of transmission occurring during the incubation period by presymptomatic individuals and by asymptomatic infections^2^. Timely quarantine of close contacts is important in preventing onward transmission during the incubation phase of the infection. In many countries, we have witnessed subsequent waves of community and household transmission leading to significant disruption, suffering, healthcare utilization, and a growing number of case-fatalities.

## 3. Seasonality of Pandemics

Multiple waves of infections are often a mainstay of pandemics (Table 1). It should be stated that when analyzing wave pattern projections for SARS-CoV-2, little data can be drawn from the other coronavirus pandemics. The 2003–2004 SARS-CoV-1 pandemic would appear as if it occurred in a single wave. However, the confinement of this virus and its limited spread to just over 8000 people make drawing parallels nearly impossible. The main thing noted in the seasonality of SARS-CoV-1 was that it appeared to spread rapidly in humans from March to June of 2003. Similarly, with MERS, this virus has had a much smaller total number of cases, was facilitated by camel to human transmission and arose in a very different environment than other pandemic viruses. The main trend seen with MERS seasonality was that between 2012–2017 the smallest number of cases were seen in July and tended to increase throughout the year, with 52.7% of all cases having occurred in April, May, and June of each year [19]. For these reasons, the discussion of seasonality and multiple waves will be limited to influenza pandemics. The timing of multiple pandemic waves is highly variable and unpredictable due to the large number of factors contributing to disease emergence and spread. Most pandemics have multiple waves peaking in non-summer months, with subsequent waves causing high levels of mortality and large variability between different regions around the globe. Although mechanisms leading to subsequent waves of infection and predicting future pandemic wave patterns are not well understood, there are a series of determinant variables we know to influence wave timing and severity. Discussed in more depth during this section, it would appear climate and environment is the major driver in seasonal timing of waves and lack of summer outbreaks. Viral (prior immunity, cross-wave immunity, mutation, etc.) and human determinants (changing population size, NPI employments, vaccines, and social changes) appear to be major drivers in the number of waves seen and help to determine the variations seen in different pandemics (Table 1).

Part of the variability of seasonality we have seen is also due to the fact that human society has been a moving target for each strain. For example, the 1889 pandemic had the first wave initiated in Europe during the end of spring in 1889 and did not peak in the United States until the beginning of 1889–1890. The lack of globalization and intercontinental travel may have influenced both the slow spread and its waves being annual [20]. This may have also been a factor as in 1889, the three resurgent spikes in strain infectivity were approximately spaced by 3 years. However, with the pandemic of 1918 and many of the pandemics since, the viral strains have had their resurgent waves follow a more annual pattern along with other seasonal flu viruses. With the exception of the severity of the 1918 H1N1 influenza pandemic, the gradual increase in travel and the global population has altered the homogeneity of waves around the world in subsequent pandemics. The SARS-CoV-2 first wave occurred within Asia, Europe, and North America within a 3-month period. For our current pandemic and future novel viruses that have a threshold transmissibility, the likelihood of global spread is elevated and is more likely to share simultaneous wave patterns than wave patterns described in influenza pandemics of the 20th and 21st centuries; and of the SARS pandemic.

The influence of seasonality has been one of the strongest and most consistent variables in wave timing and refractory periods. This is true for both seasonal pandemic influenza strains with decreased infectivity during periods with increased temperature and absolute humidity [21]. This can help to account for why most pandemic strains have shown a refractory period of decreased cases during the summer months. However, while these trends have also been seen with beta-coronaviruses, a recent study on the likelihood of SARS-CoV-2 entering into the seasonal flu community in the post-pandemic period has estimated that the influence of summer climate aspects may only decrease the likelihood of spread of SARS-CoV-2 by less than 20% [13,22,23,24,25]. While it is likely that environmental factors decrease the transmissibility of SARS-CoV-2, we are unable to say whether it occurs to the same degree as seen with influenza [22,26,27,28].

In 1968–1969, the H3N2 pandemic was the first time the United States engaged in wide-scale federal regulation of quarantine, social distancing, and personal protective equipment for both health care workers and civilians. All of these measures would have helped decrease the initial wave severity. Unfortunately, the lack of regulations allowed schools to commence following the winter break, which likely catalyzed the dual peaked first wave observed 2 months later [5]. This was by no means the only factor in the United States, as military bases and communal areas potentiated the chain of transmission. It appears that another wave of SARS-CoV-2 transmission in the United States correlates with school openings and, to some degree, to the political events and messaging prior to the elections of 3 November 2020.

## 4. Clinical Outcomes of the Different Pandemics

The pandemics discussed here span a period of over 100 years, during which advances in the biomedical and clinical landscape have improved our understanding of severe pandemic outcomes. One of the most common severe outcomes of most viral respiratory pandemics is acute respiratory distress syndrome (ARDS). Multiple mechanisms contribute to this syndrome’s progression, such as the host’s immunophenotype as well as their comorbidities [29,30]. As a result, ARDS severity and presentation can differ dramatically. Early COVID-19 studies report that 20–42% of hospitalized patients develop ARDS, subsequently some of these critically ill patients are then discharged home and expected to reintegrate into their communities. While improvements in ICU care will save many lives, many of these individuals will suffer from long-term sequelae dubbed “post-ICU syndrome” [31]. This includes reductions in their mental, cognitive, and psychological health, muscle weakness, decreased pulmonary function, and prolonged return to their activities of daily life. Health care systems must plan for the surge in post-ICU syndrome and its complications, especially for the most vulnerable groups in our community.

Secondary bacterial infections (SBI) are a well-studied severe outcome of influenza pandemics. Data indicate that, on average, between 11–35% of hospitalized patients with influenza have a bacterial co-infection, with this number being as high as 65% in immunocompromised individuals [32]. This outcome was also a likely cause of death during the 1918 influenza pandemic, prior to the advent of penicillin [33].

The role of SBIs in COVID-19 is less clear. Early reports suggested co-infections with respiratory pathogens were a rare occurrence, but recent studies have identified co-infections in up to 25.8% of hospitalized patients, with rates rising to 65% among ICU patients alone [34,35]. The majority of these were caused by viral pathogens, with bacterial and fungal infections being more common among severely ill patients [35]. Most of these studies do not provide data on the timing and development of SBIs, but the prolonged length of illness and high intubation rates may explain many of these cases. As in most pandemics, with the 2009 H1N1 pandemic being the exception, those above 65 years of age have represented one of the groups most at risk for severe outcomes, including ICU admission, mechanical ventilation, and death [3,9,36].

## 5. Mortality and Case Fatality of Pandemics

The ways in which we access mortality and case-fatality for each pandemic have changed drastically from 1918 to the present [37,38,39,40,41,42,43,44,45]. This should first be explained as direct comparisons are often difficult due to these differences in mortality parameters (Table 2). This table is by no means a definitive record of the infection and mortality rates brought about by these pandemics. These numbers are widely contested, many of the cases may not have been recorded, and the records that do exist vary greatly in coverage and reliability. 

When looking at the earliest pandemic discussed here (1918–1919 H1N1), the lack of data from both the time period and chaotic environment of WWI limit wide analysis of mortality. Much of these data are taken from retrospective studies on individual communities, areas which took a more detailed recording of the deaths and probable causes than much of the world at this time, usually documented as “total deaths”. These data are often extrapolated and overall estimates should be seen to have a very large range of error. This changed for both the 1957–1958 and 1968–1969 pandemics, where technology had progressed to the point of identifying the viral culprit during the pandemic and the implementation of better tracking and mortality data for non-pandemic years. In these cases, “excess mortality” for various age groups is the most common method. Usually using the Serfling Regression model, which compares deaths during the pandemic to pneumonia and influenza deaths in the preceding 5–10 year’s seasonal flu mortality data. The most recent pandemics tend not to use “excess mortality” to the same degree. With the advent of PCR in the late 1980′s allowing for confirmation of infection in patients and post-mortem viral detection in unknown cases, pandemics such as SARS-CoV-1 are often reported as total infections, total deaths, and age group share of total deaths (what percentage of all deaths can be attributed to each group) [23].

**Figure 1:**
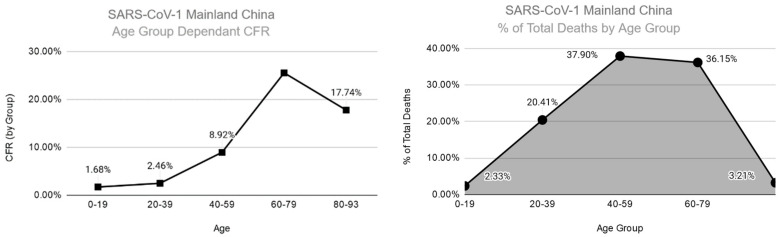
Age group dependent CFR (left column) as compared to % of total deaths for various age groups (right column) mortality (left column) for SARS-CoV-1 in Mainland China. Showing how CFR is much higher for advanced age while the majority of deaths occurred in younger populations exposed to the virus in both the community and hospitals [24].

It is also important to state that the statistics of mortality may be somewhat skewed as there is little data for any pandemic on the number of infections and deaths for equatorial countries, with the majority of data coming out of Europe, North America, Australia, and a handful of countries in Asia. This has implications as there are differences in life expectancy and the average age of populations, thus the impact each pandemic may have had is different in different areas. For the most part, understanding how these viruses affected various age groups in Europe and North America, with data from China, Japan, and South Korea coming with more recent pandemics. This can be seen with SARS-CoV-1 where it appears that the CFR and percentage of total deaths in Mainland China are higher in the 60–79 age range (25.51% CFR and 36.15% of total deaths) than in the 80–93 age range (17.74% CFR and 3.21% of total deaths) [24]. While this data looks different than in other countries, which have age and age-dependent CFR directly correlated, it may be due to the younger demographics in China and the proportionally smaller 80–93 aged population having decreased exposure in metropolitan public spaces (Figure 1). There is also a certain degree of change that happens as models progress overtime. This was seen as recently as with the 2009 H1N1 pandemic where in the years following the pandemic, the CDC has estimated the global death toll may be closer to 280,000 deaths than the 2184 lab-confirmed deaths initially reported [25]. Knowing the implications of excess mortality definitions changing over time and the somewhat limited ability to extrapolate data from a limited geographical and economic subset to the world, let us discuss the general trends seen.

**Figure 2:**
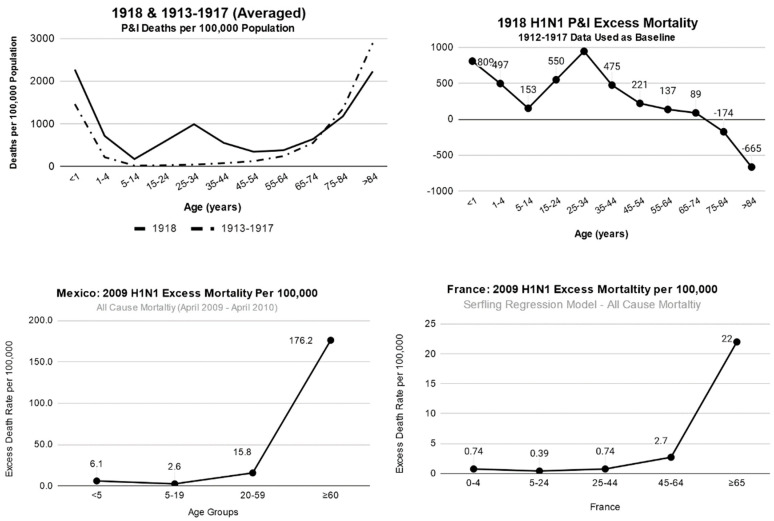
Mortality visualizations for H1N1 pandemics of both 1918–1919 and 2009. Averaged mortality (upper left) as compared to excess mortality (upper right) due to 1918 H1N1 pneumonia and influenza (P&I) pandemic. Excess death rates [5] are shown for the 2009 H1N1 pandemic for both the country of Mexico (lower left) [38] and France (lower right) [39].

For influenza, there tends to be a general trend that the two most heavily hit age groups are the very young and those over the age of 65, commonly referred to as the “U-shaped” distribution. This trend has been true for the most part for both seasonal and pandemic strains. There is, however, an exception for the H1N1 pandemics of both 1918 and 2009. In these two cases, there seems to be increased excess mortality for young adults not seen with other influenza (Figure 1 and Figure 2). This is especially true for 1918, where a “W-shaped” excess mortality curve is seen. With a number of compounding factors, young adults (15–35 years of age) had a heightened death rate (per 100,000 population) [14].

**Figure 3:**
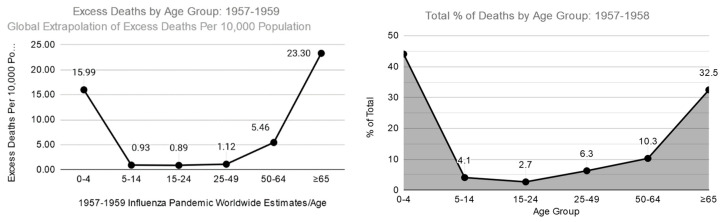
Excess deaths (right column) and total percentage of deaths (right column) due to 1957–1959 Influenza pandemic [40].

The other atypical trend seen in 1918 was that when comparing death rates to pneumonia and influenza of 1913 to 1917, people over the age of 74 had negative excess mortality [26]. A similar trend was seen in 2009 with elevated mortality of those under the age of 20 [27,28]. In both these cases, there seems to be a trend where a H1N1 related strain had been circulating decades beforehand and then gone dormant for a few decades. This provided mortality protection for older generations and increased mortality for younger generations that were not exposed to the virus during their lifetime. The 1957 and 1968 pandemic both show a more typical influenza excess mortality curve where the two most affected groups are those under the age of 4–5 and those over the age of 65, as can be seen for 1957–1959 below (Figure 3)**.** When looking at the changes from 1918 to 2009, one of the most consistent trends is that excess mortality for those over the age of 65 has remained quite high, while the excess mortality for young children has decreased with medical advances. Lastly, it is important to note that while excess mortality and the percentage of total deaths have decreased for young children, just like in 2009, these groups still maintain a large number of hospitalizations and a large total burden on the health care system.

**Figure 4:**
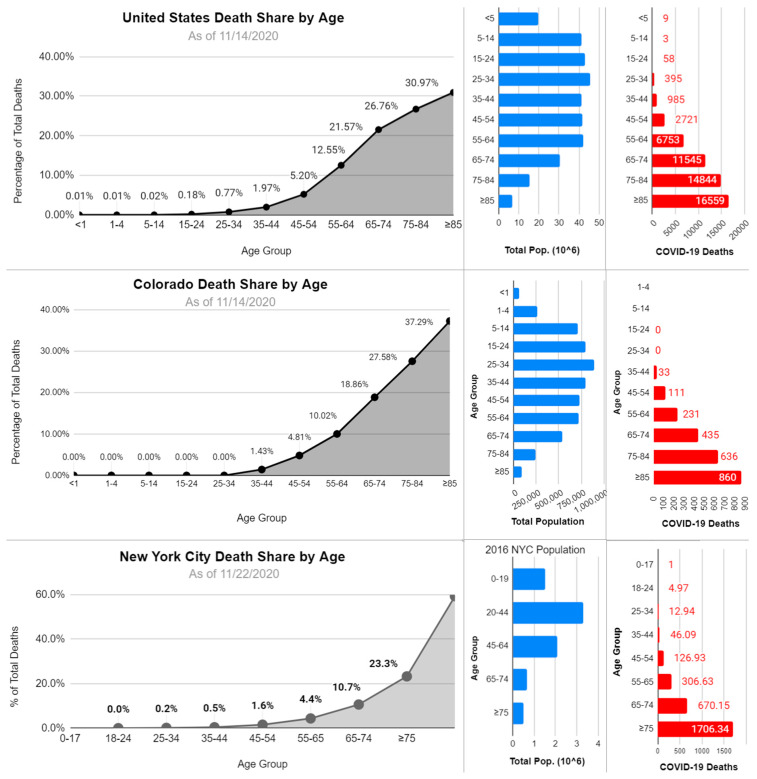
Deaths based on three regions for SARS-CoV-2, data included was last updated between 14 November–22 November 2020. The top row shows the percentage of total SARS-CoV-2 deaths (left column), regional demographics (middle column), and the total number of deaths (right column) for the United States, Colorado, and New York City. This serves to show how regional demographics can influence total death share data and can change noticeably throughout different regions. For the United States and Colorado, data were compiled from the CDC Provisional COVID-19 Data Sets [41]. For New York City, the city government’s data set was used [42].

This is quite different from Coronavirus excess mortality and total deaths. For both seasonal and pandemic CoV strains, there is a consistent trend where increases in age and mortality are directly related. When analyzing SARS-CoV-1 and MERS-CoV, there are still cases of children and young adults having some deaths. As referenced in the “Clinical outcomes of different pandemics” section, the proportion of hospitalizations and deaths increases with each decade of life, somewhat stabilizing with those over the age of 65–75. This trend is seeming to be true for SARS-CoV-2 as well, even when looking at countries with different relative age group populations. What is also noticeable about the regional data available for SARS-CoV-2 is the changes to the total % of deaths depending upon the demographics of the area. When comparing the United States, Colorado, and New York City (Figure 4), it can be shown how demographic data can often skew how the total death share appears. This is most evident in New York City, where the population demographics are noticeably younger than for the United States as whole.

## 6. Conclusions

When comparing major influenza and coronavirus pandemics in recent history, it becomes clear that their similarities are not mere coincidences but seem to be somewhat inherent to how and why certain pathogens are easily transmitted from person to person and usually by multiple modes of transmission. In the past 100 years, all major pandemics have been caused by respiratory viruses that emerged during the winter season in the Northern Hemisphere during the second half of the normal flu season. They have all originated from a non-human source and most have exhibited the ability to cross from humans back into certain animal populations serving as the site for antigenic shifts or genetic recombination events. While there is no guarantee all future pandemics will follow these trends, understanding these animal to human transmission patterns may assist us in preparing for future emerging infections. Pandemics tend to occur in at least two consecutive waves of transmission. The impact of each wave depends on the underlying level of immunity of the population and community mitigation interventions. In every pandemic, there are specific age groups at risk of developing severe disease and dying. Most of these outcomes are the result of large pools of immunologically naïve populations, immunosenescence of the elderly, and a high prevalence of medical comorbidities. Among many influenza pandemics, school-age children were major drivers of household transmission. The COVID-19 pandemic has been associated with a high-attack rate among household contacts. Therefore, the prompt institution of mitigation interventions including non-pharmaceutical interventions such as social distancing, travel restrictions, interruption of mass gatherings, and community quarantine is critical to achieving at least a 50% reduction in transmission, which correlates with a basic reproductive number lower or equal to one (R_0_ ≤ 1).

The development of vaccines is a vital part of any pandemic response. However, in all cases starting with the great influenza pandemic of 1918–1919, the timeline for vaccine development, testing, approval, and production has been inadequate to have a substantial effect in mitigating the spread and impact of each pandemic. The first 2–3 waves are most likely to occur within 12–15 months of the virus originating and therefore it is crucial to focus on mitigating interventions targeting infection prevention strategies [12]. Indeed, it is important to allocate the bulk of our public health efforts to non-pharmacologic interventions and to the medical care of symptomatic cases to reduce fatalities until safe and efficacious vaccines are fully developed and distributed. Another important aspect to consider during a pandemic is maintaining adequate coverage levels of routine childhood vaccination in order to prevent the occurrence of outbreaks of vaccine-preventable diseases.

Given the degree of globalization and interconnectivity of modernity, pandemics remain as perennial threats for human societies. We can never be fully prepared for future pandemics. However, given that pandemics tend to disproportionately impact socially disadvantaged populations, there is an important urgency to continue addressing health inequities and structural social vulnerabilities that many people globally endure.

## Figures and Tables

**Table 1:** Comparison of influenza pandemics from late 19th to 21st century.

Virus Pandemic Years	Waves (Duration)	Timing	Most Severe Wave	Post-Pandemic Resurgences	Other facts
**H3N8** **Russian Flu** **1889–1890**	3 (3 years)	W1: 1889–1890 W2: 1890–1891 W3: 1892–1893	N/A	3 each separated by 3 years. Concurrent with seasonal flu	Gradually from Asia to North America, with peak wave timing spread over months.
**H1N1** **Spanish Flu** **1918–1919**	3 (9 months)	W1: Jun–Jul 1918 W2: Oct–Nov 1918 W3: Feb–Mar 1919	2	R1: Jan–Apr 1920 R2: Dec 20–May 21 R3: Dec 21–Apr 22	W1 mostly in the US and Europe. W2 and 3 similar timing globally
**H2N2** **Asian Flu** **1957–1959**	3 (15–18 months)	W1: Sep–Dec 1957 W2: Dec–Mar 1958 W3: Dec 58–Apr 59	2	-	Midst of Vietnam war with heavy annual travel of soldiers from Asia to United States
**H3N2** **Hong Kong** **1968–1970**	2 (12–18 months)	Based on global circ. W1: Jul 68 to Aug 69 W2: Sept 69–Sept 70	2	-	Travel based spread due to the Vietnam war. US and Canada W1 most severe. All other countries W2 had highest mortality
**H1N1** **Swine Flu** **2009–2010**	1 Epidemic 2 Pandemic (12 months)	E1: Jan–Mar 2009 W1: Mar–Aug 2009 W2: Aug 09–Jan 10	2 (Fall)	-	Following the 2008 economic crisis with overflow effects on the availability of international aid

**Table 2:** Comparison of influenza and coronavirus pandemics of the 20th and 21st centuries.

Pandemic	Viral Strain	Total Infected (Globally)	Total Deaths (Globally)	Case Fatality Rate (%)	Basic Reproductive Number (R_0_)	Reference for CFR Estimates
**Spanish Flu** **(1918–1919)**	aH1N1	300–450 million	20–50 million	CMR ~2.5% *	2.11–2.5	[37]
**Asian Flu** **(1957)**	aH2N2	500,000,000	700,000–1.5 million	0.02–0.05%	1.8	[43]
**Hong Kong Flu** **(1968)**	aH3N2	500,000,000	500,000–2.0 million	0.67%	1.28–1.58	[43]
**Swine Flu** **(2009)**	H1N1pdm	200,000,000	2185–284,000	1.09%	1.33–1.38	[28]
**SARS** **(2003)**	SARS-CoV-1	8098	774	9.6%	2.4	[44]
**MERS** **(2012)**	MERS-CoV	2499	858	34.4%	1.1–1.2	[44]
**COVID-19** **(2020)**	SARS-CoV-2	58,480,000	1,385,000	2–3%	1.5–3.5	[45]

* Crude mortality rate (CMR) of ~2.5% calculated based on excess mortality observations, which may be misleading due to large geographic variations and gaps in data. Note that CMR should not be confused with Case Fatality Rate (CFR). Estimates of R_0_ were based on analysis of mortality data from the fall wave in the United States and United Kingdom.
